# A wind-tunnel gust generator for soaring birds and small UAVs

**DOI:** 10.1242/jeb.250430

**Published:** 2025-10-27

**Authors:** Matthew Penn, George Yi, Simon Watkins, Shane P. Windsor, Abdulghani Mohamed

**Affiliations:** ^1^ School of Engineering, RMIT University, Melbourne, VIC 3000, Australia; ^2^ University of Bristol, Bristol BS8 1QU, UK

**Keywords:** Gust mitigation, Bioinspiration, Avian flight, Gliding, SUAV

## Abstract

The operation of small uncrewed aerial vehicles (SUAVs) is limited by their inability to maintain steady flight trajectories in gusty conditions. Birds, however, regularly fly in the same gusty conditions with apparent ease. The mechanisms birds use to maintain steady flight in these conditions are not well understood. A wind-tunnel gust generator was developed to produce vertical and rolling gusts to perturb soaring birds so that their gust responses may be studied. The gust generator was located downstream of the birds and modified the strength of the updraft in which the birds soared. An example downward step gust, upward step gust and rolling pulse gust were characterised to demonstrate the gust generator's performance. The gusts were highly repeatable and resulted in changes of effective angle of attack of up to 20 deg, in periods of 0.2 s. Nankeen kestrels (*Falco cenchroides*) successfully soared above the operational gust generator, demonstrating its potential for use in future studies.

## Teaser

**Summary:** Development of a new wind-tunnel gust generator to study how gliding birds respond to gusts.

## INTRODUCTION

As the size of an aircraft reduces, it becomes increasingly susceptible to gust-induced perturbations (Watkins et al., 2006). Thus, small uncrewed aerial vehicles (SUAVs) are particularly prone to attitude perturbations and flight path deviations in gusty conditions (Mohamed et al., 2014). Conventional control systems lack the control authority and rapidity required to counter these perturbations effectively (Mohamed et al., 2016; Panta et al., 2018). As a result, SUAV operations are typically limited to flight in open environments with relatively calm weather conditions.

Birds share a similar range of sizes, masses and flight environments with SUAVs; however, birds appear to fly in gusty conditions with ease. The mechanisms birds use to achieve this remain little studied and poorly understood. Researchers have studied the flight of hummingbirds in gusty and turbulent conditions (Badger et al., 2019; Ortega-Jimenez et al., 2014; Ravi et al., 2015, 2020). The hummingbirds were consistently observed to respond to unsteady flight conditions by modulating the amplitude, frequency, pitch and symmetry of flapping strokes. The aerodynamics of flapping bird flight is very different to the aerodynamics of fixed-wing aircraft, however. While soaring, birds glide with their wings essentially ‘fixed’ in a similar manner to fixed-wing aircraft. Thus, the control strategies used by gliding birds are more directly relevant for engineers seeking inspiration for improving the flight path steadiness of SUAVs.

Studies focused on birds gliding in gusts are limited. Researchers have investigated several control responses employed by a steppe eagle soaring in atmospheric conditions (Reynolds et al., 2014) and a barn owl gliding through strong upward gusts (Cheney et al., 2020). The steppe eagle was observed to perform ‘wing tuck’ manoeuvres that were thought to be a gust response; however, this could not be definitively confirmed as the flow conditions the bird was responding to were not known (Reynolds et al., 2014). The barn owl was observed to elevate and pitch its wings. It was hypothesised that the wing elevation served as a preflexive (passive) response that provides rapid inertial gust damping (Cheney et al., 2020). Modelling of the wing elevation and pitching responses showed that they provided passive gust mitigation and alleviated longitudinal instability (Stanton, 2024). These studies of birds flying in gusts have resulted in several fruitful observations, but they are limited in scope to several species and specific gust conditions. There remains much to be learned by studying the flight of additional bird species in a diverse range of gust profiles, to more fully characterize the range of control strategies used by birds. One flight condition that particularly highlights birds' gust responses is windhovering.

Windhovering is a hunting behaviour used by several bird species, including kestrels (Videler and Groenewold, 1991). Windhovering birds fly into the wind at the windspeed, such that the bird remains hovering in a fixed position relative to the Earth. Kestrels have been observed hovering with their heads deviating from a fixed position by less than ±6 mm (Videler and Groenewold, 1991). With a suitable updraught, kestrels can windhover while gliding with wings fixed (Videler and Groenewold, 1991). A gliding windhover provides a unique opportunity to study birds' gust response kinematics. Propulsive kinematics (flapping) are eliminated, and the birds remain stationary throughout the gust encounter. In earlier work, nankeen kestrels (*Falco cenchroides*) were trained to windhover in an updraught in a wind tunnel (Penn et al., 2022). That work was continued in this article, with the development of a gust generator that may be used to perturb the windhovering kestrels with known and repeatable gusts.

Gusts have been defined as ‘a flow structure that exists in the environment and causes unsteadiness in the flow about a lifting surface, ultimately resulting in unsteady loading on that surface’ (Jones et al., 2022). This unsteadiness may arise as a result of changes in flow velocity magnitude, flow direction or both. Gusts that are problematic for SUAVs are caused by two main phenomena: the turbulent mixing of the atmospheric boundary layer, and the wake structures that form as wind flows around obstacles (Mohamed et al., 2023). These gusts are particularly significant at low altitudes and in rough terrains where atmospheric turbulence is most intense and obstacles are present within the flow. Gusts found in the atmosphere are highly dynamic three-dimensional flow structures; however, for research purposes, a range of simplified one- or two-dimensional profiles are typically studied.

These profiles may be grouped into three main categories: streamwise, transverse and vortical (Jones et al., 2022). Streamwise gusts refer to a fluctuation in the streamwise (*u*) component of velocity and are experienced by a flying craft as a change in airspeed. Transverse gusts refer to fluctuations in the vertical or crosswind (*v* or *w*) components of velocity and are experienced by a flying craft as a change in effective angle of attack or sideslip. Vortical gusts refer to rotational flow structures. The susceptibility of an aircraft to different types of gusts is dependent on its configuration, stability characteristics and flight condition. Generally, however, transverse gusts are more perturbing than streamwise gusts (Thompson et al., 2011), and downward and rolling gusts have been identified as particularly problematic (Mohamed et al., 2014). Thus, improving the gust-mitigating capabilities of SUAVs to downward and rolling gusts is of particular importance.

Few measurements have been taken of gusts in the real-world flight environments of birds and SUAVs. Atmospheric turbulence has been measured using pressure probes mounted to a 4 m mast above a moving car, in various wind and terrain conditions (Thompson et al., 2011; Watkins et al., 2006). The results show that 10–15 deg fluctuations in flow pitch angle are common, even above open terrain such as cleared farmland. Velocity fluctuations of 1–2 m s^−1^ were common in a measurement with an average windspeed of 4.5 m s^−1^ (Thompson et al., 2011). Gust scales in atmospheric turbulence range from tens or hundreds of metres (Flay and Stevenson, 1988; Walshe, 1972) down to millimetres (Ting, 2016). Example gust profiles have also been extracted from numerical simulations of wind structures around a nominally cuboid building (Mohamed et al., 2023). Changes in local flow velocity and direction were recorded for simulated flight paths at different airspeeds and heights above the building. The most severe gust reported was for a flight path 1 m above the building at an airspeed of 5 m s^−1^. The gust had a 20 deg change in angle of attack and a 5 m s^−1^ change in airspeed occurring in 0.25 s. The gust characteristics dampen with increased flight velocities and greater elevation above the building. Similar flow conditions may be encountered above topographic features such as cliffs. While limited, these examples give some indication of the gust magnitudes that birds and SUAVs may encounter.

Existing gust generation methods are not well suited for perturbing live birds windhovering in a wind tunnel. Past experiments involving free-flying birds (Badger et al., 2019; Bamford et al., 2024; Cheney et al., 2020; Quinn et al., 2019), insects (Jakobi et al., 2018) and model aircraft (Bamford et al., 2024; Donely, 1939; Oduyela and Slegers, 2014) have required the test subject fly through transverse jets of air. This is unsuitable for windhovering where the birds remain hovering in a fixed location. This may be overcome by integrating transverse jets into a wind-tunnel test section (see for example Ryan and Dominy, 1998; Smith 2018). Modulating the jets on and off can introduce gusts; however, this also introduces complex wake patterns into the flow that reduce gust repeatability. Alternatively, gusts are commonly generated using upstream oscillating louvres (Brion et al., 2015; Mankowski et al., 2014; Saddington et al., 2015; Wood et al., 2017) or other lift-generating devices (Ham et al., 1974; Tang et al., 1996; Umbarger, 1970). A test subject may be precisely positioned between louvre wake structures such that smooth flow is maintained throughout the gust encounter. This is suitable for a sting-mounted model (Hamada et al., 2019; Kobayakawa and Maeda, 1978; Ricci and Scotti, 2008; Wu et al., 2013), but infeasible for a live bird in free flight. Because of the disadvantages associated with existing gust generation methods, a novel gust generator to perturb soaring birds and SUAVs was developed for this work. The objective of this paper is to describe the function of the gust generator, characterise the flow field for example gusts and demonstrate its function in flight tests with live kestrels.

List of symbols and abbreviations

GR: gust ratio
SUAV: small uncrewed aerial vehicle
*t*_e_: encounter period
*u*: streamwise component of velocity
UAV: uncrewed aerial vehicle
*v*: lateral component of velocity
*V*: absolute velocity
*w*: vertical component of velocity
*W*_e_: encounter width
*W*_g_: gust width
α: angle of attack
β: sideslip angle
Δ: change in
θ: flow pitch angle
ѱ: flow yaw angle

## MATERIALS AND METHODS

### RMIT Industrial Wind Tunnel

The gust generator was developed for use in the RMIT Industrial Wind Tunnel. The tunnel is closed circuit, has a 2:1 contraction ratio, and a test section measuring 9 m long, 3 m wide and 2 m tall. A flow-smoothing screen was installed, resulting in a turbulence intensity of 0.8% with an empty test section.

### Dynamic updraught gust generation concept

**Fig. 1.**
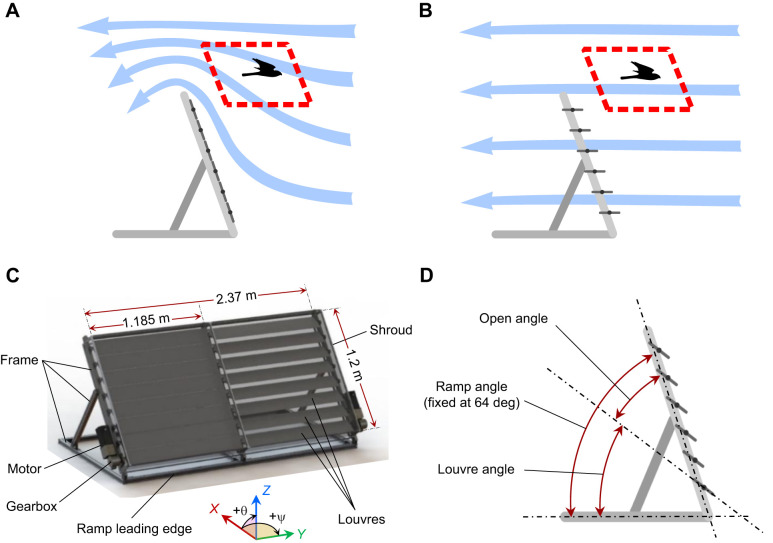
**The gust generation device.** (A,B) A schematic diagram of the gust generation concept. When the louvres are closed (A), the flow is forced over the gust generator, causing an updraft. When the louvres are opened (B), the flow passes between the louvres, reducing or eliminating the updraft. The target flight region is highlighted with the dashed red line. (C) A drawing of the gust generator. The louvres on the left side of the image are fully closed, while the louvres on the right side are partially opened. Direction conventions used throughout the article for position (*X*, *Y*, *Z*) and flow angle (pitch θ and yaw ѱ) are also indicated. (D) The angle definitions used throughout this article.

The gust generator was designed to integrate with an existing wind tunnel setup which had been developed to study the flight of soaring birds and SUAVs in smooth flow and well-mixed turbulence. This facility is described in greater detail in Penn et al. (2022). A ramp was used to generate an updraft, simulating orographic updrafts found in nature. The gust generator replaces the ramp's surface with banks of louvres. These louvres may be rotated to adjust the strength of the updraft produced. When the louvres are closed, the air is forced over the gust generator, producing an updraft in the same manner as an orographic updraught. This is illustrated in Fig. 1A. As the louvres are opened, some of the flow passes between the louvres, reducing or eliminating the updraft, as illustrated in Fig. 1B. Dynamically opening or closing the louvres allows updraft strength to be reduced or increased, respectively, generating downwards or upwards gusts. The gust generator has two independently activated banks of louvres located side by side. Vertical transverse gusts may be produced by activating both banks together, while rolling gusts may be produced by generating an upward gust on one side and a downward gust on the other.

This unusual gust generation concept possesses several key advantages for the study of birds. It integrates seamlessly into the flight environment in which birds have already been trained to soar, and it generates downward and rolling gust profiles, both of which are known to be particularly challenging for SUAVs. The gust generator is also located downstream of the test subject. This provides a large volume free of obstacles in which the bird or SUAV may safely fly, and it maintains smooth flow by eliminating wake structures in the target flight region. A disadvantage of this method is that the gust has a combination of streamwise and transverse components, and gust characteristics are dependent on location relative to the gust generator.

### Gust generator construction

A drawing of the gust generator is shown in Fig. 1C. The device has two banks of eight louvres, forming a total surface area 2.84 m^2^. Each louvre has a chord length of 0.15 m, a length of 1.185 m and a thickness of 0.018 m. The chord length was chosen to balance the competing objectives of reducing motor torque requirements (favouring shorter chord lengths with lower moment of inertia) and minimising manufacturing complexity (favouring larger chord lengths with fewer, more robust louvres). The louvres were constructed with a fibreglass shell around a foam core and a central steel spar.

The gust generator was built to fill as much of the test-section width as possible, while still leaving a narrow walkway (<0.3 m) to one side to allow the bird handler to access birds both upstream and downstream of the gust generator. A space of 0.17 m on either side of the gust generator was also required for the frame and motor mounting.

Each bank of louvres was independently actuated by a Beckhoff AM8051-1-0E11-0000 servomotor with a 7.5:1 reduction gearbox (Beckhoff Automation Pty Ltd, Mount Waverly, VIC, Australia). The motors were powered by a Beckhoff AX5206 driver and controlled using TwinCAT XAE 3.1 software. The lowest louvre in each bank was driven directly by the motor, and the remaining louvres were coupled via a mechanical linkage.

A safety shroud was constructed from a coarse plastic mesh and secured to a light aluminium frame. The mesh had 25 mm spacing and an openness ratio of 82%. The shroud was fitted to both the front and rear surfaces of the gust generator, completely encasing all moving parts.

The frame was constructed from aluminium extrusions with a 45×45 mm cross-section. The base of the frame was anchored to the floor of the test section. The upper portion of the frame housed the louvres, motors and shroud. The upper portion of the frame was hinged at the leading edge, allowing the angle of the ‘ramp’ formed by the frame to be adjusted.

The terms used to describe the angle of the frame and louvres throughout this article are defined in Fig. 1D. The ramp angle was fixed at 64 deg for the duration of the testing in this paper, as this was found to generate a suitable updraft for soaring birds, while also allowing both upward and downward gusts to be produced. The angle of the louvres is called the ‘louvre angle’ when defined relative to the horizontal, and the ‘open angle’ when defined relative to the frame of the gust generator.

### Louvre deflections

Louvre motion was controlled by specifying the desired deflection magnitude, as well as the maximum values of velocity, acceleration and jerk. Two deflections could be separated by a delay to produce pulse and top-hat gust profiles, and the gust generator could be programmed to repeat a predefined sequence of different gust profiles. Adjusting these parameters allowed a wide range of deflection profiles to be achieved. This study focused on producing sharp-edged step and pulse gust profiles to perturb the gliding birds. Simplified gusts such as these are desirable for studying the birds' fundamental control responses.

Maximum deflection rates achieved by the motors depended on wind loading and deflection magnitudes. For the profiles tested in this study, the maximum angular velocities achieved were 9.8e2 deg s^−1^, maximum angular accelerations were 7.7e4 deg s^−2^ and maximum angular jerk values were 6.3e6 deg s^−3^. This corresponds to a louvre deflection of 30 deg in 0.1 s, or a 5 Hz cycle. The freestream windspeed was 5.5 m s^−1^, which is a suitable speed for windhovering kestrels.

**Fig. 2.**
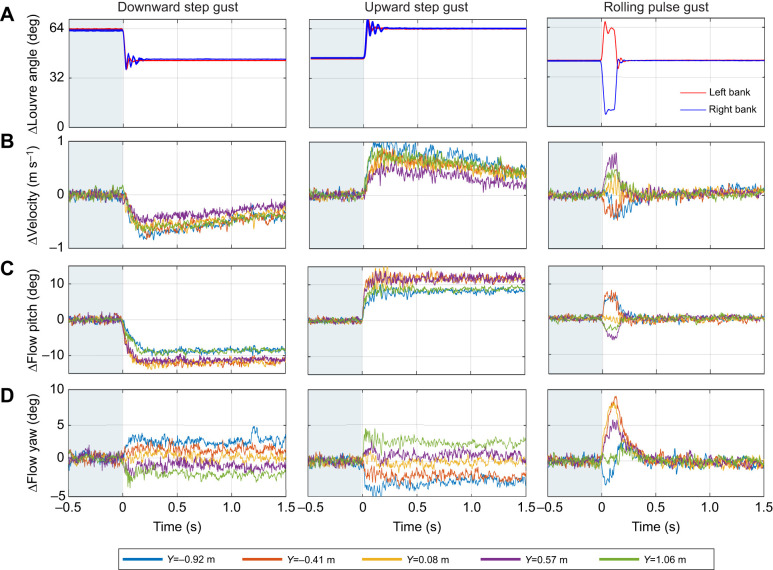
**Gust time-history profiles.** (A) Louvre deflection profiles for three example gusts. Gust activation begins at *t*=0 s. (B–D) Time-history flow measurements for these louvre deflections: change in velocity (B), flow pitch (C) and flow yaw (D). Change in flow parameters was calculated by subtracting the mean value from *t*=0 to 0.5 from the signal. Measurements were taken once at *X* and *Z* coordinates of 7.37 and 1.4 m.

Three gust profiles are used as examples in this article to demonstrate the gusts produced by the gust generator. The louvre deflections for the three gusts are plotted in Fig. 2A. The first gust is a downwards step gust, where both banks of louvres begin at a louvre angle of 64 deg (fully closed) and simultaneously deflect to a louvre angle of 44 deg (−20 deg deflection). The second gust is an upward step gust where louvres begin at a louvre angle of 44 deg and deflect to 64 deg (fully closed). The third gust profile is an asymmetric pulse of the louvres, causing a rolling gust. Both banks of louvres begin at a louvre angle of 44 deg. The left bank deflects to 64 deg (+20 deg deflection), while the right bank deflects to 14 deg (−30 deg deflection). The result is an upwards gust on the left and a downwards gust on the right, causing a moment that would roll a bird or aircraft to the right. These three examples demonstrate both step and pulse gusts, as well as the ability of the gust generator to produce downwards and rolling gusts which are reported to be the most difficult for SUAVs to counter (Mohamed et al., 2014).

### Flow measurements

A flow mapping exercise was undertaken to map the static and dynamic flow fields around the gust generator in several example gust conditions. Mapping of the flight volume was conducted by using arrays of point measurements from a Cobra Probe (Turbulent Flow Instrumentation Pty Ltd, Tallangatta, VIC, Australia). Cobra Probes measure the three orthogonal velocity components *u*, *v* and *w*. Cobra Probes were calibrated by the manufacturer. Post calibration probe velocity measurements are accurate to within ±0.3 m s^−1^ and angle measurements are accurate to within ±1 deg at typical test velocities (https://www.turbulentflow.com.au/Products/CobraProbe/CobraProbe.php). Cobra Probe measurements have previously been validated in various flow conditions (Chen et al., 2000; Hooper and Musgrove, 1997; Mousley et al., 1998). Cobra Probes have a cone of acceptance of ±45 deg, and data falling outside this range were rejected. During testing, zero samples were recorded outside this range, so all data recorded were deemed reliable. Measurements were recorded at 2.5 kHz, then resampled at 300 Hz to match the sample rate of the motion tracking cameras described below.

**Fig. 3.**
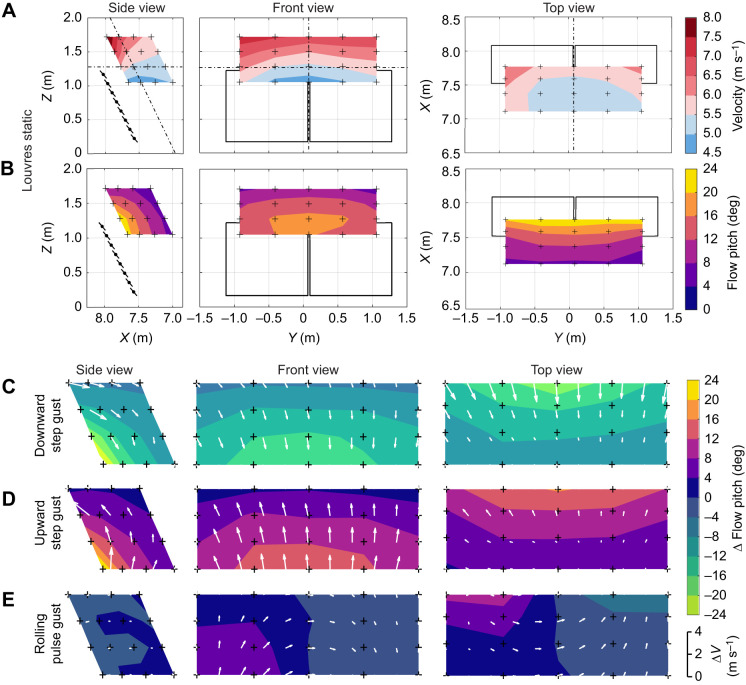
**The flow field in the flight volume is visualised with contours on three intersecting planes.** (A) The velocity magnitude distribution and (B) the flow pitch angle distribution when the louvres are held static at 49 deg (15 deg open). The position of the planes relative to the gust generator is visualised with the dashed lines in A. Measurements were taken once at the locations marked by ‘+’. Note that the plane on which the front view is plotted is inclined at the ramp angle of the gust generator (64 deg). Plot boundaries in A and B show the test section walls and ceiling, except for the ‘*X*’ direction which is 9 m long. Freestream wind flows in the +*X* direction, and positive pitch angle is defined as flow with a +*Z* velocity component. (C–E) Peak gust magnitudes and directions for the three example gusts. Arrows indicate the change in local flow velocity, and are scaled according to the key in the bottom right of the figure; they do not represent the wind direction.

The Cobra Probes were mounted on a 2-axis traverse, with the vertical axis inclined to match the ramp angle. The inclined vertical axis (*Z* direction) was automated and actuated by a stepper-motor, while the longitudinal axis (*X* direction) was manually adjusted. The entire traverse was removed and reinstalled laterally within the test section to measure multiple lateral positions (*Y* direction). Thus, measurements were taken to form an oblique three-dimensional grid. The measurement points are indicated by the crosses in Fig. 3. At each location, measurements were taken to record the flow field with louvres held statically and with a range of louvre deflection profiles. The static flow field was mapped with the louvres set to an open angle of 15 deg (a louvre angle of 49 deg), and a freestream flow velocity of 5.5 m s^−1^, which was found to be suitable for soaring kestrels through flight testing.

### Gust characterisation metrics

Simplified transverse gust profiles may be well characterised by three metrics – magnitude, scale and development rate (Stutz et al., 2023). Gust magnitude may be evaluated using the change in velocity Δ*V* (or the three orthogonal velocity components Δ*u*, Δ*v*, Δ*w*). Gust magnitude is often non-dimensionalised by dividing by the unperturbed velocity *V* to find the gust ratio GR, as per Eqn 1 (Stutz et al., 2023).
(1)

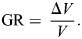
For transverse gusts, gust magnitude may also be expressed as change in flow pitch (Δθ) and yaw (Δѱ). This convention is preferred throughout this article, as the aerodynamic effects of changes in flow angle are more intuitively understood. Changes in flow pitch and yaw will be experienced by an aircraft as changes in local angle of attack (Δα) and sideslip (Δβ). Gust magnitudes were calculated as the difference between the mean flow values in periods before and after each louvre deflection. For each event, the mean before the deflection was obtained from 50 samples (0.17 s) collected immediately before the deflection. The mean after the deflection was obtained from 50 samples collected in the interval *t*=0.67 to 0.83 s for the step gusts and 25 samples in the interval *t*=0.52 to 0.60 s for the pulse gust. The sample sizes were chosen to balance two considerations: maximising mean accuracy by increasing the number of samples, and minimising the influence of low-frequency variations in the gust profile by shortening the sampling period. Sample timing was selected manually to capture the period when each gust was at its peak.

Gust scale is quantified using the gust width *W*_g_ and encounter period *t*_e_. *W*_g_ may be non-dimensionalised to the encounter width *W*_e_ by dividing by the chord length *c* of the test subject, as shown Eqn 2 (Stutz et al., 2023):
(2)

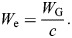
In this work, the start (and end) of *t*_e_ was defined as the time when Δθ exceeded (and returned to) an angle of 1 deg from the initial condition. *W*_g_ was calculated from *t*_e_ and local flow velocity *V* using Eqn 3 (Stutz et al., 2023):
(3)

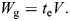


*W*_g_ for the gust generator may be modulated by adjusting the delay time between the louvres opening and closing (thus modulating *t*_e_). Increasingly, large *W*_g_ may be produced by lengthening the delay, and vice versa. There is no upper limit to the length of *W*_g_ that may be produced. A step gust theoretically has infinite *W*_g_ (Stutz et al., 2023). Minimum *W*_g_ is determined by the deflection rates of the louvres as well as the local velocity.

Gust development rate refers to the rate of change of the gust magnitude, and may be expressed in a variety of ways, e.g. 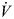, 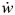, 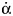, etc. Before development rates were calculated, the time-history gust signals were smoothed with a 4th order low-pass Butterworth filter to eliminate high-frequency velocity fluctuations due to noise. A 20 Hz cutoff frequency was found to effectively smooth the signal while retaining the gust profile. See Fig. S1 for an example. Development rates were then calculated by taking the numerical time derivative of the gust signal. Peak development rates were reported, as the development rates vary with time. The 90% rise time (time from | Δθ |>1 deg to GR>0.9×GR_max_) was used to quantify the period required for the step gust magnitudes to plateau.

### Flight testing

Flight tests with two live nankeen kestrels (*Falco cenchroides* Vigors & Horsfield 1827) were conducted to validate the gust generator's ability to perturb soaring birds. Both birds were female and raised in captivity. The birds were believed to be 7 and 14 years old, but their exact ages were unknown. The flight tests were approved by the RMIT University Animal Ethics Committee (AEC 2022-24233-17592, approved 2 June 2022) and the Victorian Government's Department of Environment, Land, Water and Planning (Permit No. 10010385, approved 22 June 2022).

During flight testing, the freestream wind was set to 5.5 m s^−1^ when the louvres were set to a louvre angle of 49 deg (15 deg open). The birds were trained to windhover above a small box on the floor containing a food reward. After a successful flight, the box was remotely opened allowing the kestrel to claim its reward.

**Fig. 4.**
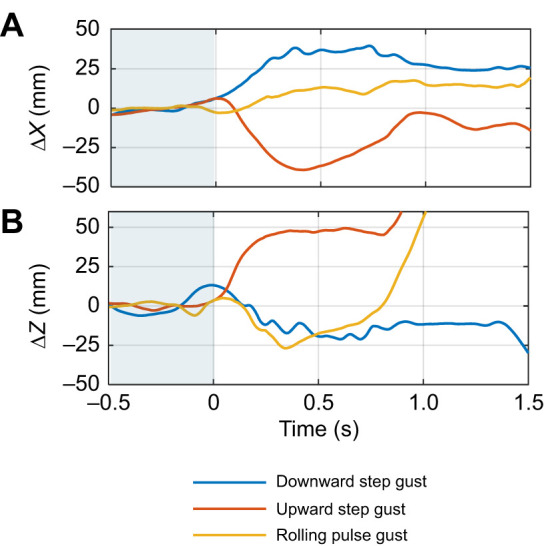
**Time-history signals showing displacement of the kestrels' body position throughout three sample gust encounters.** (A) Displacement in the *X* (freestream, horizontal) direction and (B) displacement in the *Z* (vertical) direction. Videos of these gust encounters are shown in Movies 1–3. Gust activation begins at *t*=0 s. The mean value of the signals between *t*=0 and *t*=0.5 has been removed.

Gusts were generated using a wide range of louvre deflection profiles to see how the birds would respond to different gusts. Three sample videos of these tests are provided (Movies 1–3), and the displacements of the kestrels' body positions throughout these gust encounters are plotted in Fig. 4. Movies 1 and 2 show encounters with the downward and upward step gusts characterised in Figs 2 and 3. Movie 3 shows an encounter with a rolling pulse gust, where both banks of louvres began at a louvre angle of 64 deg (fully closed). The right bank deflected to a louvre angle of 14 deg (50 deg open), then returned to the initial position, while the left bank of louvres remained stationary.

### Motion tracking

Flight kinematics and louvre deflections were recorded with Qualisys motion-capture cameras (Qualisys AB, Göteborg, Sweden). Twelve Oqus 7+ infrared cameras and three Miqus M5 optical cameras were distributed around the edges of the test section ceiling. The motion-capture system was calibrated to within 0.4 mm. Kinematics were captured at 300 Hz, and optical recordings were captured at 25 Hz. Fifty-one reflective markers were attached to the birds' feathers using double-sided adhesive tape. Markers with 1.5 mm diameter were used at the tips of the flight feathers, while hemispherical markers with 3 mm diameter were applied to the covert feathers and the shafts of flight feathers inboard from the tip. The kestrels' body position was tracked using 3 mm markers adhered to the bird's mantle feathers. The deflections of the upper-most louvre on each side of the gust generator were tracked with 4 mm spherical markers applied to the leading and trailing edges.

### Manuscript preparation

ChatGPT-4 was used during manuscript preparation to improve grammar and clarity, to generate title and summary statement suggestions, and to review the reference list for errors. The authors subsequently reviewed and edited the content as necessary and take full responsibility for the publication's final content.

## RESULTS AND DISCUSSION

### Flow field with louvres fixed

The flow around the gust generator is three-dimensional, with wind velocity (both magnitude and direction) varying in a continuous manner throughout the volume. This is visualised in Fig. 3A,B, which shows the velocity and flow pitch angle distributions on three intersecting planes or ‘slices’ through the measurement volume when the louvres are held fixed at 49 deg (15 deg open). Fig. 3A shows that the flow velocity increases from the freestream, reaching a maximum above the top lip of the ramp, due to the flow contraction. Fig. 3B shows that flow pitch angle is greatest close to the louvres and decreases as distance to the gust generator increases. The gaps between the test section walls and the edges of the gust generator have a significant effect on the flow structure. The flow of air around the edges of the gust generator causes an increase in local flow speed (Fig. 3A) and a reduction in local updraft angle (Fig. 3B). This is more pronounced on the side of the gust generator where the gap is larger (left side of the top view in Fig. 3A,B). Changing the angle of the louvres changes the flow field. Increasing the louvre angle results in a reduction in freestream velocity (due to increased blockage); however, the flow pitch angle increases.

### Gust magnitude and direction

Fig. 3C–E shows the gust magnitude and directions for the three example gusts. The change in flow pitch (Δθ) corresponds to the change in effective angle of attack (Δα) for a hovering bird. Δα is arguably the most important measure of gust amplitude, as changes in angle of attack have far greater impact on lift production than changes in streamwise velocity (Thompson et al., 2011). Δθ is visualised by the contours in Fig. 3C–E. Although peak Δθ for the step gusts reaches >±20 deg, most of the flight volume Δθ values range between ±4 and 12 deg. As with the static flow field, the gust magnitude is not uniform throughout the flight volume. Δθ diminishes rapidly as distance upstream from the gust generator increases. For the symmetric gusts, Δθ is greatest in the centre of the gust generator and diminishes gradually towards the edges as a result of the gaps between the gust generator and test section walls. The symmetric step gust has Δθ values double those of the asymmetric pulse gust, even though the louvre deflection magnitudes are similar. Comparison with additional gust profiles in Fig. S2 reveals that this difference was due to the asymmetric deflection rather than the pulse.

The arrows in Fig. 3C–E indicate the direction and magnitude of changes in flow velocity. For the symmetric gusts (Fig. 3C,D), the gust direction is predominantly streamwise at the top of the flight volume, but transitions to predominantly transverse at the bottom of the flight volume. There is also an increase in flow yaw towards the edges of the gust generator due to the gaps between the gust generator and test section walls. The asymmetric gust (Fig. 3D) introduces rotational motion into the flow, with both rolling and yawing rotational components. The updraft side of the gust generator has greater change in effective angle of attack (and transverse velocity), while the downdraft side of the gust generator has a more prominent change in streamwise velocity. The central region of the flight volume experiences a predominantly yawing transverse gust.

This variation throughout the volume means that the gust characteristics encountered by a bird or SUAV will depend on the hover location. The gust encountered may be predominantly a change in velocity magnitude, flow pitch or flow yaw. Also, a bird or SUAV that is not centred on the gust generator may experience a rolling moment even when louvre deflections are symmetric, as the wing closer to the centre of the gust generator may experience a larger change in effective angle of attack than the outer wing. In many cases, the tail surfaces of a bird or SUAV would experience larger magnitude gusts than the main wing (as a result of closer proximity to the gust generator). These effects would be amplified for larger aircraft.

### Gust development rate

Fig. 2B–D shows time-history measurements for the three gust profiles taken at different lateral locations across the span of the gust generator. Maximum gust development rates for the three example gusts are listed in Table S1. Of the two symmetric gust profiles, the upward step gust develops more rapidly than the downward step gust, despite the similarities in gust magnitudes. Both step gusts develop more rapidly than the rolling pulse gust.

The transverse velocity components of the step gusts accelerate more rapidly than the streamwise gust components. This difference is visually noticeable in the much sharper change in flow pitch angle than the change in absolute velocity in Fig. 2B,C. This difference between 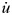 and 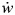 is not pronounced in the pulse gust.

Gust development rates reduce significantly as streamwise (*X*) distance from the gust generator increases. Table S2 lists the peak development rates for step and pulse gusts at different streamwise locations. The development rates decline at roughly 50% per 0.25 m. From visual inspection of the gradients in Fig. 2B–D, it is evident that the gusts at the edges of the gust generator have lower development rates in the pitch (*Z*) direction, but more rapid development in the streamwise (*X*) and yaw (*Y*) directions.

Gust development rates vary with time. For the step gusts, 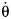 initially increases sharply, then reduces with time until the flow pitch stabilises at a new value. The 90% rise times are 0.09 (downward) and 0.07 s (upward). After the step gusts, flow pitch plateaus at a steady value; however, flow velocity peaks before slowly declining. It takes approximately 10 s to stabilise at a new value. It probably takes this long because of the flow in the whole wind tunnel needing to stabilise after a rapid change in blockage.

### Gust width

The minimum gust period (*t*_e_) for a 20 deg louvre pulse deflection was 0.12 s, which corresponds to an effective gust width (*W*_g_) of 0.72 m with a local flow velocity (*V*) of 6.0 m s^−1^. The chord length of kestrels' wings (*c*) is approximately 0.1 m; thus, the minimum encounter width (*W*_e_) for flight tests with the kestrels is 7.2. This value represents the sharpest pulse gusts that may be generated. In practice, flight tests were often conducted with larger gust widths. *t*_e_≈0.2 for the rolling pulse gust in Fig. 2, corresponding to a *W*_e_≈12. During flight testing, step gusts were separated by roughly 4 s, resulting in *W*_e_ values of several hundred.

Larger gust widths are generally more perturbing than smaller gusts. If *W*_e_<1, then only a portion of the wing is influenced by the gust at one time. Larger gusts (*W*_e_>1) immerse the entire wing in the gust flow field, resulting in larger peak gust loads (Jones et al., 2022). Larger gusts also increase *t*_e_, resulting in greater impulse on the bird or aircraft. Increasing *t*_e_ will thus increase perturbations, until some limiting *t*_e_ is reached where the bird or aircraft has returned to equilibrium through either active corrections or passive stability. From the sample flights in Fig. 4 it appears that stabilisation time for the kestrels in the example gusts could be of the order of 0.4 s (*W*_e_≈24); however, this requires analysis of a larger dataset to confirm.

### Repeatability

Gust repeatability was tested by comparing 10 repetitions of the same gusts. This was done with the downwards step gust that has already been shown in this paper, as well as its upwards step gust counterpart. The gusts were activated in a 2 s cycle. An ensemble average was taken of the 10 gusts. The gusts were highly repeatable. The average standard deviation was 0.05 m s^−1^ for velocity magnitude, 0.42 deg for flow pitch angle and 0.39 deg for flow yaw angle. These values did not vary throughout the gust cycle and were consistent with the 0.8% turbulence intensity of the tunnel in smooth flow. Thus, the variation between gust repetitions is minimal and may be fully accounted for by the tunnel's baseline turbulence intensity.

### Kestrel gust responses

The kestrels were able to successfully soar above the gust generator and made noticeable movements of their wings and tail in response to the gust encounters. Prominent responses include flapping in response to the downward step gust (Movie 1) and folding the wings and tail in response to the upward step gust (Movie 2). Perturbations in the hover positions are also evident in Fig. 4B. The upward gust causes the bird to drift backward and heave upward, while the downward and rolling gusts cause the kestrel to drift forward and heave downward. The flight tests thus demonstrated the suitability of the gust generator to facilitate the study of birds' gust response kinematics. Analysis of the kestrels' gust response kinematics will be the subject of further study.

### Comparison with gusts in nature

Table 1 compares the characteristics of the gusts in this work with the characteristics of transverse gusts recorded elsewhere in the literature. The table includes gusts measured and simulated in natural world conditions. The gusts produced in this work have similar maximum changes in flow pitch angle (≤20 deg) to those recorded in atmospheric turbulence (Thompson et al., 2011; Watkins et al., 2010) and flow structures around buildings (Mohamed et al., 2023). Gust periods (and step-gust rise times) in this work are ≥0.1 s. This encompasses the 0.25 s period for flight around the building (Mohamed et al., 2023), while atmospheric turbulence contains a wide range of gust time and length scales. These similarities show that the gusts produced in this work fall within the range of gust magnitudes and scales that birds and SUAVs are expected to encounter when flying in natural circumstances.

**Table 1.** A comparison of the gust properties produced in this work, gusts found in nature, and gusts produced by gust generators in the literature, including those used with flying birds

Gust source	Bird species	Δθ (deg)	*t*_e_ (s)	*W*_e_	Reference
Downstream louvres	Nankeen kestrel (*Falco cenchroides*)	≤20 (typically ≤12)	≥0.12	≥7	This work
Atmospheric turbulence	N/A	≤15	Widely variable	N/A	Thompson et al., 2011; Watkins et al., 2010
Simulated flow around building	N/A	≤20	≥0.25 (step rise time)	N/A	Mohamed et al., 2023
Transverse jet	Anna’s hummingbird (*Calypte anna*)	∼80	0.07	∼3	Badger et al., 2019
Transverse jet	Barn owl (*Tyto alba*)	22 to 35	0.16	∼10	Cheney et al., 2020
Transverse jet	Red-tailed hawk (*Buteo jamaicensis*)	31 and 49	0.3	∼2	Bamford et al., 2024
Upstream louvres	N/A	≤13	≥0.07	N/A	Wood et al., 2017
Upstream louvres	N/A	≤12	0.5	N/A	Saddington et al., 2015
Upstream louvres	N/A	≤12	0.1	N/A	Grissom and Devenport, 2004

The major difference between the gusts in this work and those in nature is that gusts in the natural world are complex and 3-dimensional (Mohamed et al., 2023), while the gusts produced in this work follow simplified profiles that are known, repeatable and controlled. The streamwise gust component in this work (<1 m s^−1^) is roughly half that reported for atmospheric turbulence (Thompson et al., 2011), and roughly 20% of that reported for the maximum strength gust around a building. While this differs from the gusts found in nature, minimising the streamwise gust component is helpful for isolating the bird's responses to the transverse gusts. Simplifying the flow conditions is better for identifying and understanding the fundamental control mechanisms used by birds.

### Comparison with existing gust generators

Table 1 also lists the characteristics of the gusts produced by several different gust generators in the literature, including those used in experiments with birds. Transverse-jet devices can generate gusts with very large amplitudes (limited by jet power); however, the gust width (and thus period) is limited by the width of the jet and the rate the jet is traversed. Table 1 reveals that louvre-deflection devices are limited to a maximum gust magnitude of approximately 12 deg; however, the gust periods (and thus gust widths) vary widely, depending on the motor's programming and capabilities.

The gust generator in this work generated gusts with magnitudes equivalent to the maximum produced by the other louvre-deflection devices in Table 1, but much lower than those produced by the traverse-jet devices used in studies with other birds. The gust magnitudes are deemed sufficient, however, given the noticeable responses observed in the kestrels as observed above, and the similarity of the gust magnitudes to those reported in nature.

The minimum period and width of the gusts in this work is mid-range compared with the other devices in Table 1. This is sufficient for flight tests with kestrels. The preliminary flight test results show that the gusts are rapid enough to produce observable responses. Further reducing gust period leads to a reduction in perturbation magnitude (as discussed in Materials and Methods). The ability to produce large-width gusts (such as step gusts) allows the gust generator in this work to facilitate studies that explore gust conditions not yet reported elsewhere in the literature.

The gust generator developed in this work possesses several unique advantages for facilitating studies with gliding birds. It provides a continuous updraught for sustained soaring, and a large flight volume free from obstructions. Positioning the louvres downstream also minimises visual cues associated with louvre deflections that the bird could use to anticipate gust perturbations. The safety shroud and matte-black paint further obscure visual perception of louvre deflections from the birds' hovering location.

The gust generator is also well suited to a range of additional applications as well as studying the gust responses of gliding birds. Features that allow the gust generator to facilitate gliding birds would also facilitate gliding SUAVs. It can generate the gust types (downward and rolling) that are most problematic for SUAVs (Mohamed et al., 2014). The gusts are highly repeatable as smooth flow is maintained throughout the gust encounter, and the gusts are highly customisable. For example, the gust generator may be programmed with an irregular (yet repeatable) sequence of louvre deflections to simulate the irregularity of gusts in atmospheric turbulence. The gust generator could also be programmed to simulate the flow conditions encountered in specific scenarios, such as flying through the shear layer over a building, as in Mohamed et al. (2023). This may be particularly useful during the development of gust-resistant SUAVs with autonomous orographic soaring capabilities, where large-scale gusts have proven problematic (Watkins et al., 2015). The gust generator can also be used to generate gusts for sting-mounted models or SUAVs in powered flight. This allows the gust generator to be used in a wide range of research studying gust mitigation in small aircraft.

### Conclusions

A novel gust generator was developed to perturb gliding birds. The flow around the gust generator was characterised for two gust conditions, demonstrating the gust generator's functionality. It was found that: (1) the gust generator creates flow conditions adequate to sustain soaring kestrels; (2) the gust generator produces upward, downward and rolling gusts; (3) gusts are highly repeatable as smooth flow (<1% turbulence intensity) is maintained throughout the gust encounter; (4) the gust profiles are highly customisable, allowing step, pulse, top-hat and other complex gust profiles to be produced; (5) changes in angle of attack up to 20 deg were achieved; (6) the gusts generated consisted of a combination of streamwise and transverse gust components; and (7) the gust characteristics vary significantly with location in the flight volume.

The gust generator shall be used in future studies investigating the control strategies used by hovering birds to mitigate gust disturbances.

## Supplementary Material

10.1242/jexbio.250430_sup1 Supplementary information
