# Supplementary material for: A wind-tunnel gust generator for soaring birds and small UAVs
Source: J Exp Biol. 2025 Oct 27;228(20):jeb250430. doi: 10.1242/jeb.250430 (PMC12633735; doi:10.1242/jeb.250430)
Supplement: Supplementary information [file jexbio-228-250430-s1.pdf]

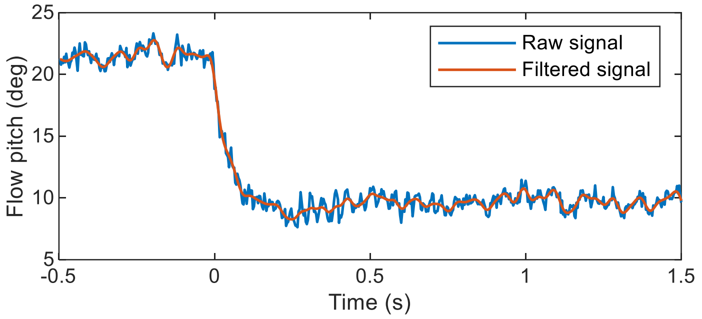

Fig. S1. A sample gust flow pitch signal smoothed with a 4th order low pass Butterworth filter with a 20 Hz cutoff frequency. The signal comes from the downward step gust in Fig 2, measured at X, Y, and Z coordinates of 7.59, 0.08, and 0.128 m.

Journal of Experimental Biology • Supplementary information

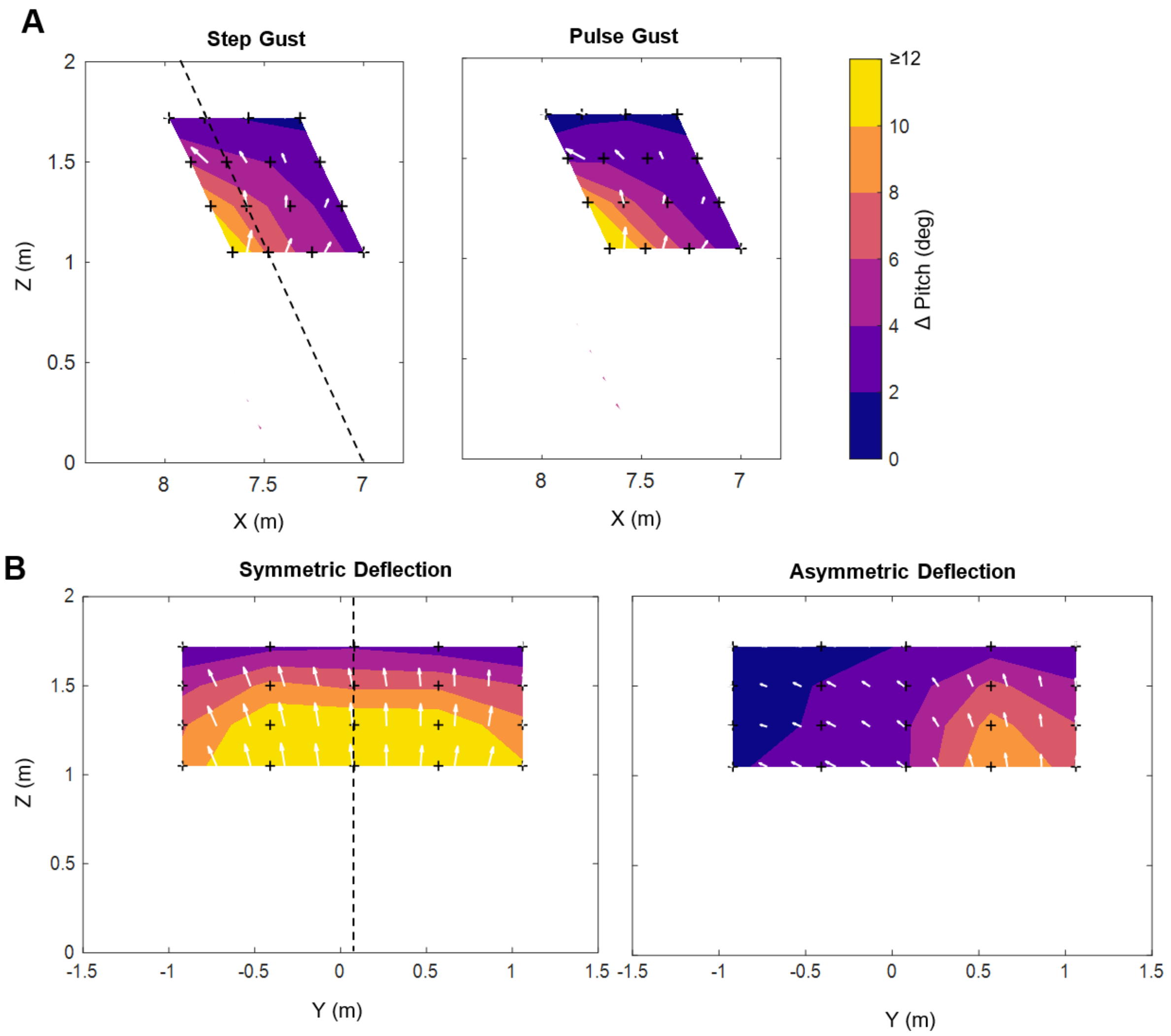

Fig. S2. A comparison of the change in flow pitch for upward gusts shows that step and pulse gusts have similar magnitudes, while gusts produced by asymmetric louvre deflections have smaller magnitudes than those produced by symmetric deflections. (A) Contours are plotted from the side view at the central plane of the gust generator (Y = 0.08 m). Both gusts have symmetric louvre deflections, where the louvres begin at a louvre angle of 49 deg (15 deg open) and deflect to a louvre angle of 64 deg (fully closed). (B) Louvres were deflected from a louvre angle of 44 deg (20 deg open) to 64 deg (fully closed). Both louvres were deflected symmetrically for the contour on the left, but only the right louvre was deflected for the contour on the right, while the left remained static. Contours are plotted from the front view on the plane shown by the dashed line in the left graphs.

Table S1. Peak gust development rates for the three example gusts. Measurements were taken in the centre of the gust generator, where X, Y and Z values were equal to 7.37, 0.08 and 1.4 m. Peak development rates below 8 m s^{-2} and 30 deg s^{-1} are unreliable due to signal noise.

Gust Type	$\lvert\dot{u}_{max}\rvert$ (m s^{-2})	$\lvert\dot{v}_{max}\rvert$ (m s^{-2})	$\lvert\dot{w}_{max}\rvert$ (m s^{-2})	$\lvert\dot{V}_{max}\rvert$ (m s^{-2})	$\lvert\dot{\theta}_{max}\rvert$ (deg s^{-1})	$\lvert\dot{\psi}_{max}\rvert$ (deg s^{-1})
Downward Step	4.0	3.3	-18.5	8.5	209	25
Upward Step	9.1	0.1	35.4	10.5	360	39
Rolling Pulse	4.3	14.8	2.3	4.2	12	151

Table S2: Peak gust development rates for the example step gust in the streamwise (u) and transverse (w) directions. Gust development rate reduces as distance upstream of the gust generator increases (-X). Measurements were taken in the centre of the gust generator, where Y and Z values were equal to 0.08 and 1.4 m. Peak development rates below 8 m s^{-2} are unreliable due to signal noise.

	Downward Step		Upward Step	
X Position [m]	$\lvert\dot{u}_{max}\rvert$ (m s^{-2})	$\lvert\dot{w}_{max}\rvert$ (m s^{-2})	$\lvert\dot{u}_{max}\rvert$ (m s^{-2})	$\lvert\dot{w}_{max}\rvert$ (m s^{-2})
7.77	5.2	34.2	15.8	67.5
7.59	5.7	18.5	9.2	35.4
7.37	7.1	6.5	8.1	18.2
7.11	6.3	5.4	6.3	11.7

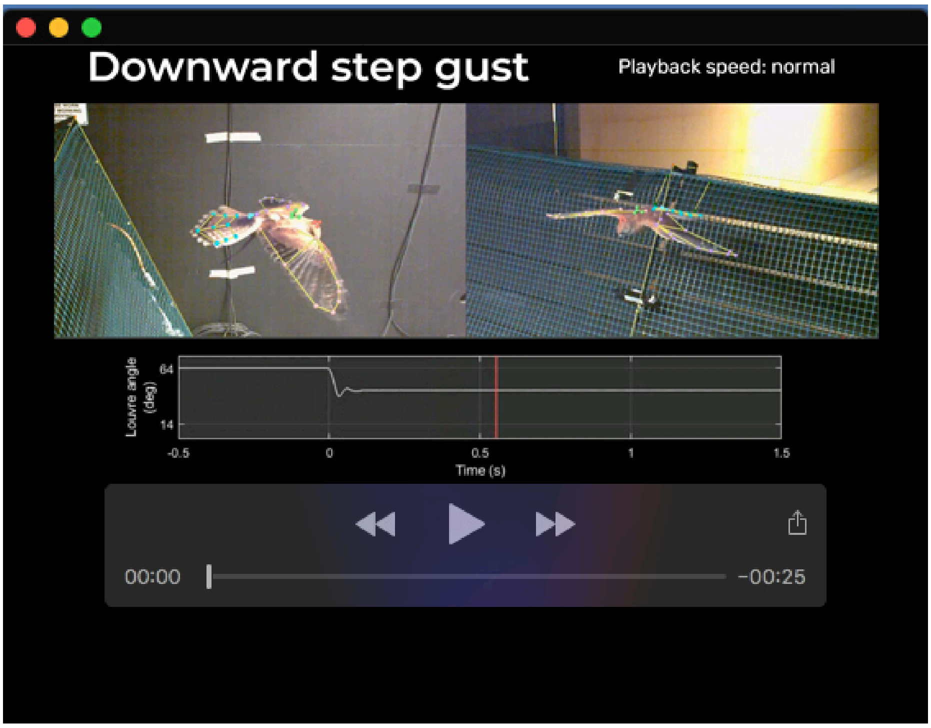

Movie 1.

Journal of Experimental Biology • Supplementary information

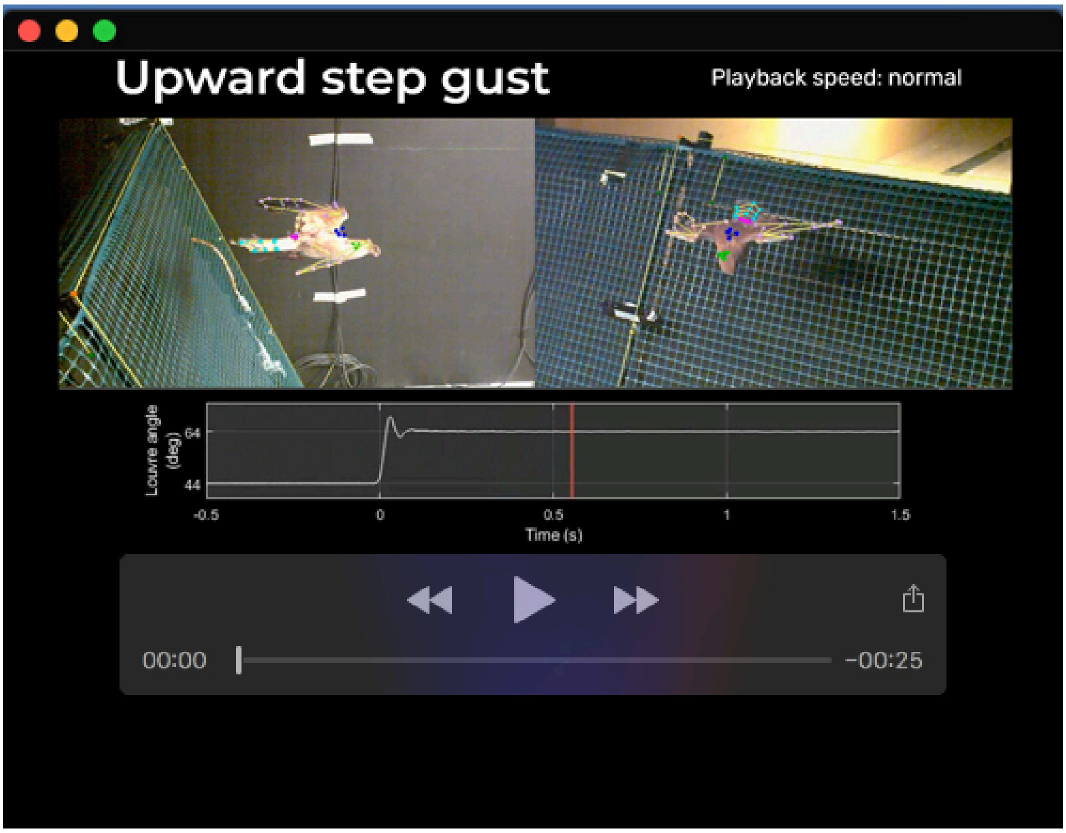

Movie 2.

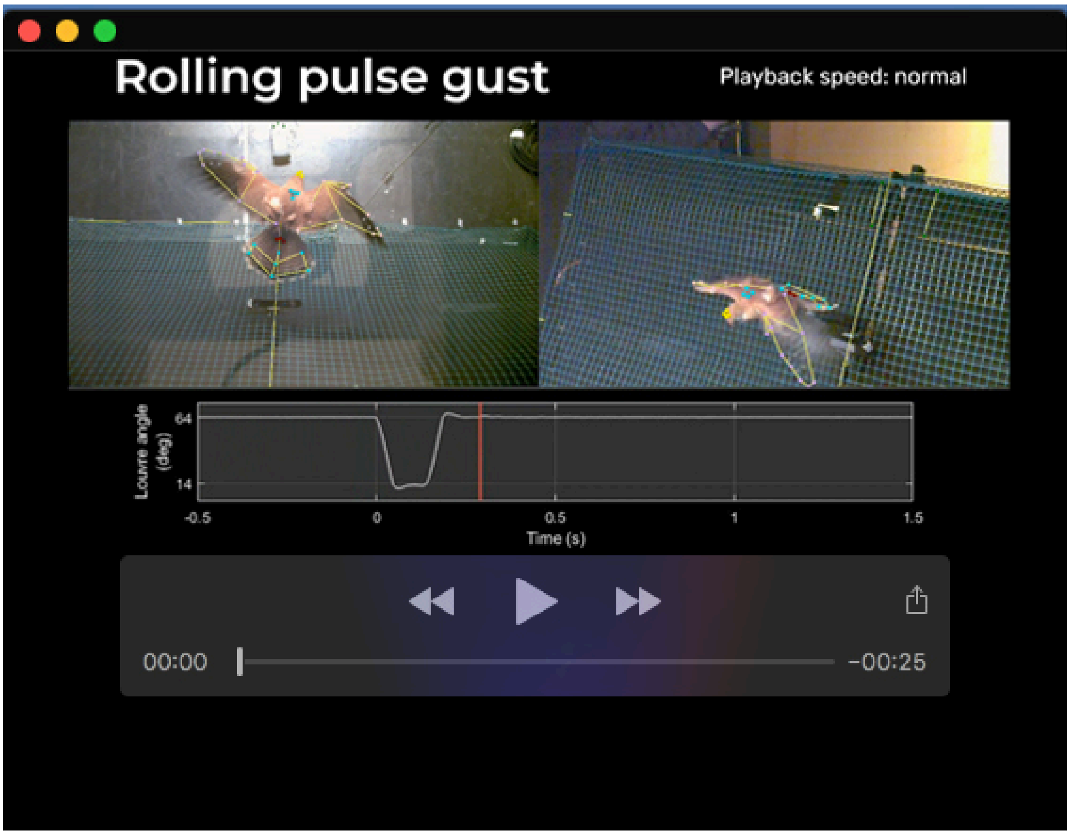

Movie 3.

Journal of Experimental Biology • Supplementary information
